# The treatment of primary and metastatic renal cell carcinoma (RCC) with image-guided stereotactic body radiation therapy (SBRT)

**DOI:** 10.2349/biij.3.1.e6

**Published:** 2007-01-01

**Authors:** BS Teh, C Bloch, M Galli-Guevara, L Doh, S Richardson, S Chiang, P Yeh, M Gonzalez, W Lunn, R Marco, J Jac, AC Paulino, HH Lu, EB Butler, RJ Amato

**Affiliations:** 1 Department of Radiation Oncology, The Methodist Hospital, Houston, Texas, United States; 2 Department of Neurosurgery, The Methodist Hospital, Houston, Texas, United States; 3 Department of Pulmonary Medicine, The Methodist Hospital, Houston, Texas, United States; 4 Department of Orthopedic Surgery, The Methodist Hospital, Houston, Texas, United States; 5 Department of Genitourinary Oncology, The Methodist Hospital, Houston, Texas, United States; 6 Department of Radiology, The Methodist Hospital, Houston, Texas, United States; 7 Department of Radiology, Baylor College of Medicine, Houston, Texas, United States; 8 Department of Pulmonary Medicine, Baylor College of Medicine, Houston, Texas, United States

**Keywords:** Renal cell carcinoma (RCC), primary and metastatic RCC, Image Guided Radiation Therapy (IGRT), Stereotactic Body Radiation Therapy (SBRT)

## Abstract

**Purpose:**

Brain metastases from renal cell carcinoma (RCC) have been successfully treated with stereotactic radiosurgery (SRS). Metastases to extra-cranial sites may be treated with similar success using stereotactic body radiation therapy (SBRT), where image-guidance allows for the delivery of precise high-dose radiation in a few fractions. This paper reports the authors’ initial experience with image-guided SBRT in treating primary and metastatic RCC.

**Materials and methods:**

The image-guided Brainlab Novalis stereotactic system was used. Fourteen patients with 23 extra-cranial metastatic RCC lesions (orbits, head and neck, lung, mediastinum, sternum, clavicle, scapula, humerus, rib, spine and abdominal wall) and two patients with biopsy-proven primary RCC (not surgical candidates) were treated with SBRT (24-40 Gy in 3-6 fractions over 1-2 weeks). All patients were immobilised in body cast or head and neck mask. Image-guidance was used for all fractions. PET/CT images were fused with simulation CT images to assist in target delineation and dose determination. SMART (simultaneous modulated accelerated radiation therapy) boost approach was adopted. 4D-CT was utilised to assess tumour/organ motion and assist in determining planning target volume margins.

**Results:**

Median follow-up was nine months. Thirteen patients (93%) who received SBRT to extra-cranial metastases achieved symptomatic relief. Two patients had local progression, yielding a local control rate of 87%. In the two patients with primary RCC, tumour size remained unchanged but their pain improved, and their renal function was unchanged post SBRT. There were no significant treatment-related side effects.

**Conclusion:**

Image-guided SBRT provides excellent symptom palliation and local control without any significant toxicity. SBRT may represent a novel, non-invasive, nephron-sparing option for the treatment of primary RCC as well as extra-cranial metastatic RCC.

## INTRODUCTION

Renal cell carcinoma (RCC) is traditionally considered to be radio-resistant and the conventional dose fraction size of 1.8-2.0 Gy is thought to have little role in the management of primary RCC especially in terms of cure. In the setting of metastatic RCC, conventional radiotherapy has been an effective palliative treatment in approximately 50% of patients [1]. More importantly, brain metastases from RCC have been successfully treated with stereotactic radiosurgery (SRS) with local control rates of more than 85% [2-5]. The advances in technology and physics in radiation oncology have led to the clinical implementation of image-guided radiation therapy (IGRT) and body stereotaxis. Thus, it is now possible to deliver very high and biologically potent dose to the tumours extra-cranially. Therefore, primary RCC as well as RCC metastases to extra-cranial sites may be treated with similar success using stereotactic body radiation therapy (SBRT), where image-guidance and stereotaxis allow for the delivery of precise high-dose radiation in a few fractions [6]. This paper reports the authors’ initial experience with SBRT in the management of primary and metastatic RCC.

## MATERIALS AND METHODS

### Patient population

This is a retrospective study of sixteen patients (fourteen patients with metastatic RCC and status of post initial nephrectomy, and two medically inoperable patients with co-existing primary and metastatic RCC) treated at a single institution. All patients signed an informed consent prior to the simulation and delivery of SBRT. The twenty-three extra-cranial metastatic RCC lesions in the fourteen patients included the orbits, head and neck, lung, mediastinum, sternum, clavicle, scapula, humerus, rib, spine and abdominal wall. The two patients with biopsy-proven primary RCC were not candidates for nephrectomy because of multiple medical problems including cardiac and pulmonary morbidity. In addition to the primary RCC, they also had metastatic RCC involving various extracranial sites. All sixteen patients with metastatic RCC involving extracranial sites were referred for radiotherapy because of local symptoms especially pain. All of these patients have received some prior systemic treatment regimens consisting of IL2, interferon, various chemotherapeutic agents, targeted therapy (such as sorafenib and sunitinib), clinical trial drugs or any combinations. They did not receive any concurrent systemic treatment with SBRT. Both of the medically inoperable patients for nephrectomy were referred because they were not candidates for any systemic treatment of either IL2 or interferon. Neither sorafenib nor sunitinib was approved for use by FDA at that time. They both had pain in the flank originating from the primary tumour. The concern was that the primary RCC may progress and cause more pain as well as deterioration of renal function.

### SBRT simulation, target delineation, treatment planning and delivery

#### IGRT linear accelerator/simulation

**Figure 1:**
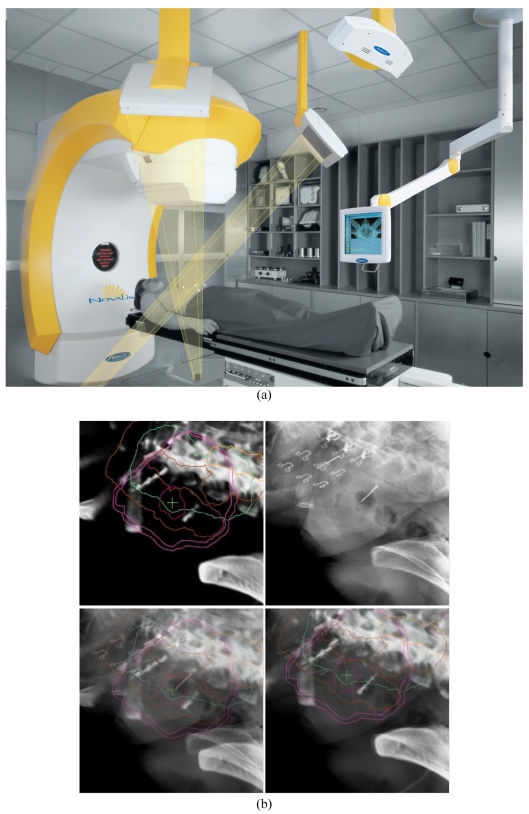
(a) The Brainlab Novalis stereotactic linear accelerator includes two orthogonal diagnostic x-ray tubes and flat panel imagers to provide image-guided 3D patient alignment. (b) Example of Novalis image alignment. Upper left panel is DRR showing expected image (from one of the two imagers). Upper right panel shows actual X-ray. Lower left panel shows overlay of the DRR and X-ray image. This information from the two orthogonal X-ray systems plus full CT data set for computation of DRRs allows calculation of patient shift to produce correct alignment. The post-shift image overlay is shown in the bottom right panel. Note the quality of the image alignment both by the bony landmarks and the implanted fiducial markers.

**Figure 2:**
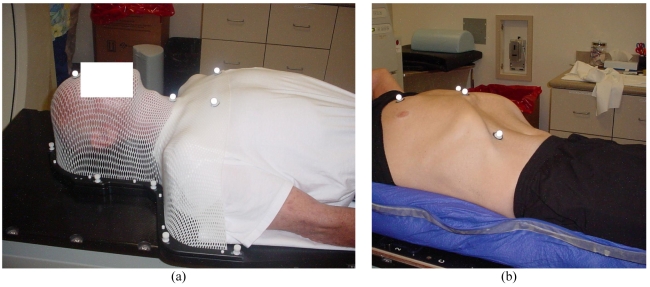
(a) Patient immobilisation using an aquaplast head and neck mask. Reflective markers on the mask are utilised by the Novalis infrared tracking system for both initial patient alignment as well as incremental shifts. (b) Patient immobilisation using a vacuum body mold. Reflective markers are again used by the infrared tracking system, which can also monitor patient motion during treatment.

SBRT is made possible with the technological advances in image-guided radiation therapy (IGRT). SBRT makes use of the principles of stereotactic radiosurgery (SRS) to provide accurate and precise delivery of high-dose radiation to targets in extracranial sites. The Brainlab Novalis system is an image-guided, shaped-beam radio-surgical unit, capable of using conformal arcs and intensity-modulated radiation therapy (IMRT). It utilises stereoscopic X-ray based localisation technology for high-precision non-invasive SBRT to extracranial targets. (Figure 1a) More specifically, the unit has the ability of corroborative image fusion of the digitally reconstructed image from CT simulation and orthogonal X-ray imagery taken in the treatment position. (Figure 1b) The robotic couch allows automated positioning of the patients to the best match of the stereoscopic images to the planning CT. Verification images are taken after the patient is aligned. Shifts of less than 1 mm are ignored. The unit is also equipped with a non-invasive, frameless positioning device that uses infrared, passive marker technology as well as the micromultileaf collimator for radiation intensity modulation and beam shaping. Using dynamic shaped beam, this system maximises the dose to irregularly shaped lesions, while minimising the dose to the surrounding normal tissues. During simulation, the patients were immobilised in a head and neck mask (Figure 2a) or body cast (Figure 2b) depending on the sites irradiated. A PET/CT was performed with the patient in the immobilisation device during the same simulation session to facilitate the optimal fusion with the simulation CT. 4D-CT was also performed to evaluate tumour and organ motion on selected patients.

#### Target delineation /organ motion/fiducial markers

SBRT target delineation generally included only gross tumour volume (GTV) as evidenced clinically and on imaging studies. The inclusion of PET/CT images to the Novalis BrainScan further refines the target delineation. MR images were also fused to aid target delineation especially in the head and neck, and spine regions. There was no true clinical target volume (CTV) representing sub-clinical disease involvement that follows the ICRU 50 paradigm. The integration of PET/CT images also allows for differential fraction size prescription/delivery, i.e. SMART (simultaneous modulated accelerated radiation therapy) boost first started in the authors’ institution [7]. This approach is also known as simultaneous integrated boost (SIB) in other institutions.

Maximum intensity projection (MIP) images from 4D-CT scans were used for delineating targets in the lung. For many patients, tumour/organ motion data were obtained using 4D-CT with patients immobilised in the body cast. There was only minimal motion with bony lesions including spine and pelvis when the patients were placed in the supine position. This was confirmed by doing 4D-CTs on the first few bony metastasis patients. These bony lesions were readily targeted for SBRT because of minimal motion and easy visualisation with the Novalis stereoscopic X-rays. For anatomical sites without good bony landmarks (e.g. kidney and liver), the placement of radio-opaque markers e.g. Visicoils^TM^ (RadioMed Corporation, Tyngsboro, MA) helps in measuring motion and providing image guidance during treatment delivery.

The size of lesions treated varied from less than 10 cc to over 200 cc. Table 1 shows the distribution of size for GTVs.

**Table 1:** Distribution of GTV size

**GTV (cc)**	**Number of lesions**
0-10	3
10-40	11
40-80	5
80-150	3
150-200	2
>200	1

GTVs (or internal target volume (ITV), including internal motion as determined by 4D CT) were expanded 2-3 mm uniformly to account for setup error. One exception was vertebral lesions that were not expanded into the cord space so that the spinal cord could be spared. A rib lesion was expanded 5 mm; image-guided setup error could be larger for peripheral targets, and no critical structures were put at risk by this expansion. Expansions were also limited when they became non-physical, e.g. if planning target volume (PTV) would extend outside the patient. One lesion received no expansion because the GTV was already larger than ideal for SBRT (280 cc).

#### Treatment planning and delivery

Individualised tumour/organ motion data obtained using 4D-CT contributed to PTV. Multiple fields (5-12) of either dynamic conformal arcs or intensity-modulated radiation therapy (IMRT) were used to maximize the treatment conformality to the tumour and the avoidance of normal tissues. IMRT is preferred for target sites that showed minimal motion such as osseous sites. Both coplanar and non-coplanar approaches have been used. Dose constraints have been placed on partial organ volume based on existing protocols and published literature [16-19], e.g. 700 cc liver receiving 15 Gy or less in three fractions [16], no more than 10% of the adjacent spinal cord receiving 10 Gy [17], no part of the esophagus, stomach or small bowels receiving 8 Gy or more per fraction, central tracheo-bronchial trees or large vessels receiving no more than 8 Gy per fraction as well as V20 of the lung is 10-15% [19]. All the SBRT planning and QA was performed by two board-certified medical physicists. The treating radiation oncologist and medical physicist were both present to ensure the most optimal image-guidance with kV-stereoscopic images overlaid on the digitally reconstructed radiographs (DRR) before each SBRT fraction.

Dose regimens are dependent on tumour volume and constraint of normal tissues. This is the initial experience with most prescriptions of 8 Gy x 3 fx, with a few exceptions. Dose escalation trials are ongoing. One patient received 8 Gy x 1 fx as a boost to previously treated (spine) lesions. Another patient with 3 small lesions (2.7, 1.9 and 40 cc) received 14 Gy x 1 fx to one lesion and 12 Gy x 1 fx to each of the other two. As discussed elsewhere, one patient received a 4 Gy x 3 fx concomitant boost to the PET-positive region of the primary kidney tumour. The last two patients in this series received higher doses, 8 Gy x 4 fx.

Generally 95-100% of the GTV received the full prescription dose. The exception was the single fraction boost treatments (8 Gy x 1 fx), where coverage had to be compromised because of the prior dose to the spinal cord. Dose homogeneity was fairly high. Again, with three exceptions GTV minimum dose (to a single voxel) was at least 93% of the prescription, while “hot” spots ranged from 103% to 112%. Naturally, PTV coverage was not as good, but with small margins and fairly homogeneous dose distributions, was not much different than the GTV coverage.

## RESULTS

**Figure 3:**
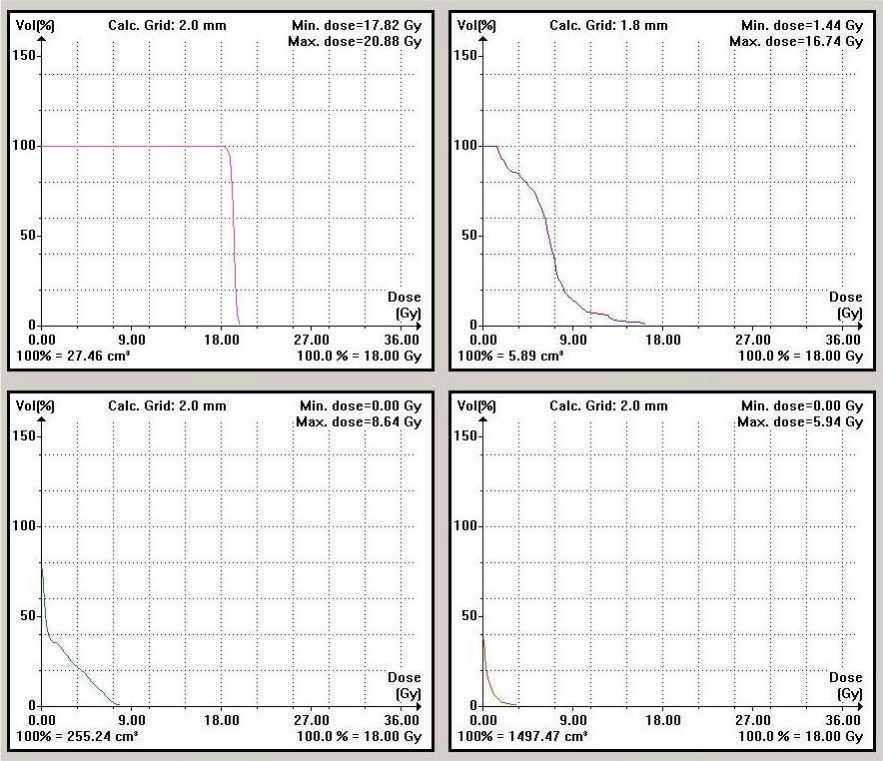
Dose-volume histograms (DVHs) from a spine treatment plan. Upper left panel shows target coverage (18Gy prescribed dose). Upper right panel shows DVH for the spinal cord near the target (from 6mm superior to 6mm inferior to target). Bottom panels show other organs at risk, left kidney (left) and liver (right).

**Figure 4:**
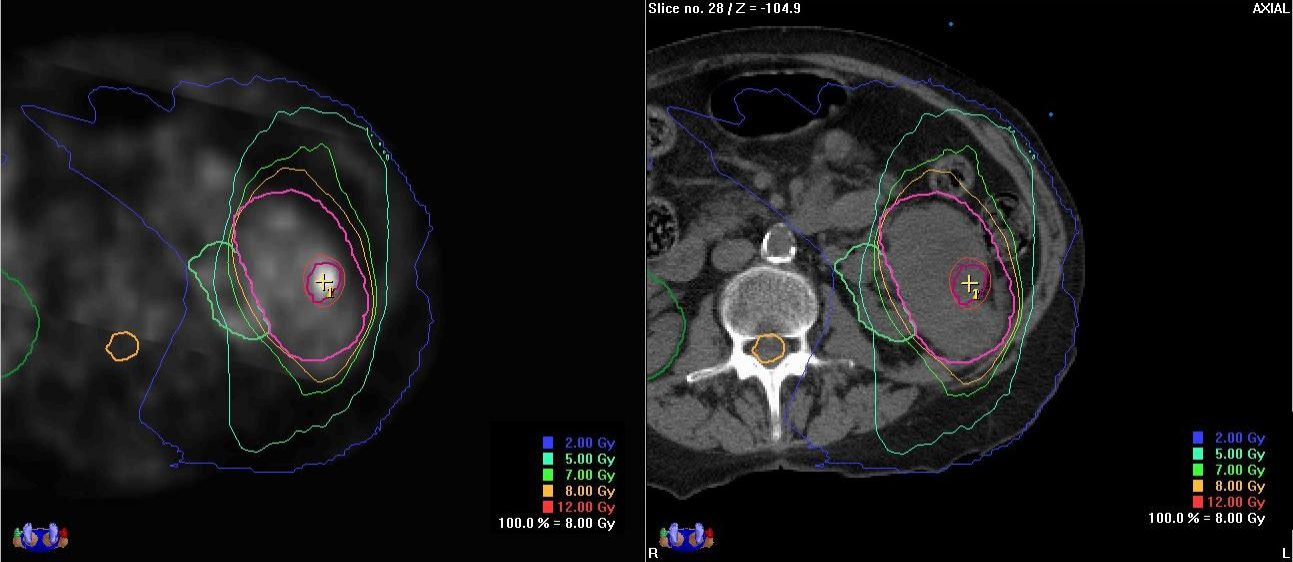
Treatment plan with SMART boost. 8 Gy prescribed to renal mass, with 12 Gy (total) going to volume with high PET activity (shown on left).

**Figure 5:**
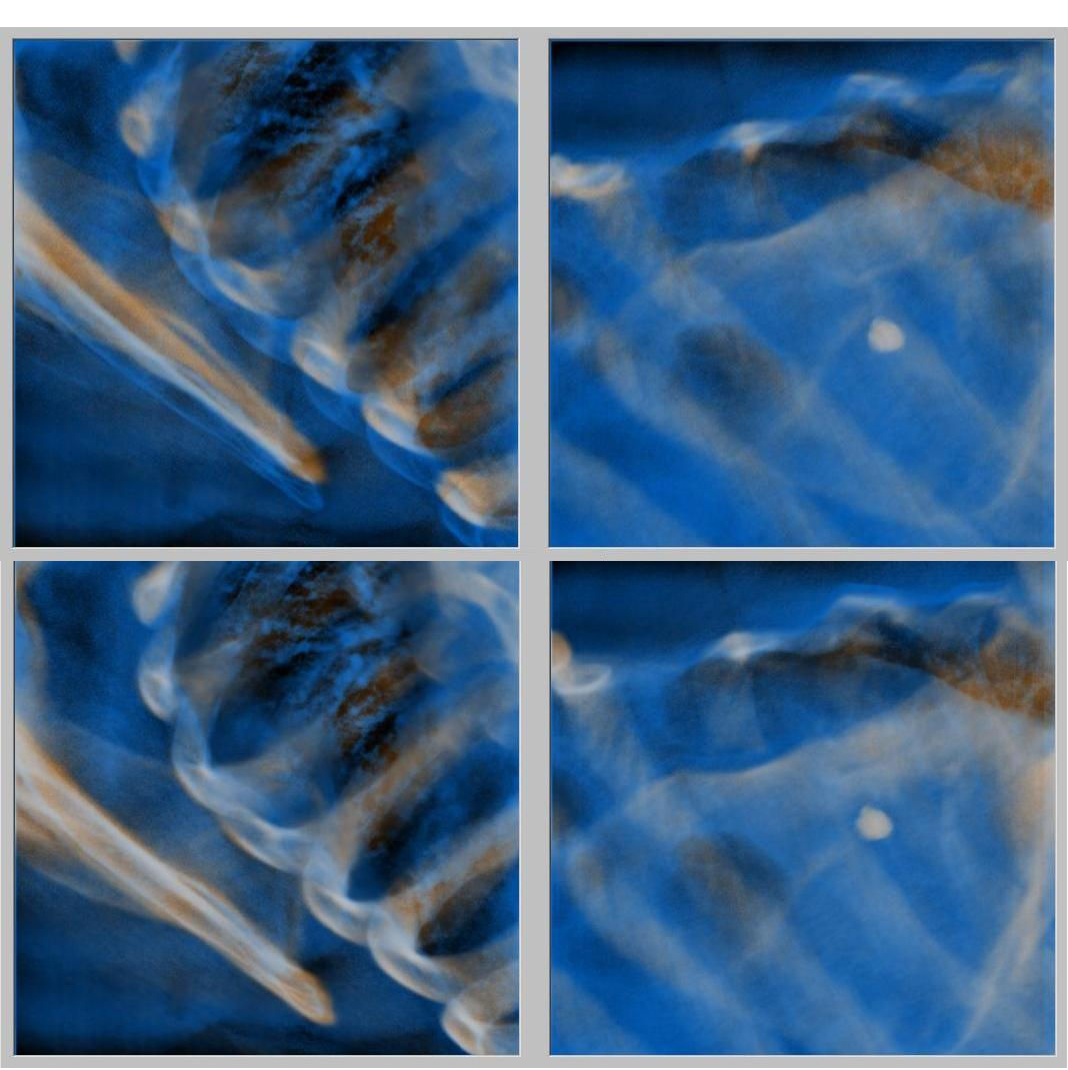
Pre- and post-alignment images for treatment of rib metastasis. Top panels show overlay of orthogonal X-rays (blue) with their expected DRRs (amber). Bottom panels show final alignment based on bony anatomy.

All of the patients were treated with SBRT using the Novalis stereoscopic X-ray based system. Median follow-up was nine months. The dose ranges from 24 to 40 Gy in 3-6 fractions over 1-2 weeks. A representative SBRT dose-volume histogram (DVH) as shown in Figure 3 demonstrates the high dose to the targets while minimising the dose to the critical surrounding structures. In comparison to the very high dose received by the tumour target in the spine, normal tissues (spinal cord, kidney and liver received very low dose because of the rapid fall-off. Figure 4 illustrates the SMART boost approach: PET avid area received a higher fraction size (12 Gy) while the rest of the mass on CT (non-avid on PET) received lower fraction size (8 Gy) to total doses of 36 Gy and 24 Gy respectively in three fractions. Note the rapid fall-off in the isodose lines. Figure 5 illustrates a bony site treated with SBRT and the image-guidance stereoscopic X-rays that show excellent alignment.

### Patients’ tolerance

All of the patients were treated in the supine position. The treatment time for SBRT is longer when compared to that of the conventional radiotherapy because of set-up, image-guidance and re-alignment as well as multiple treatment fields and larger monitor units for high radiation dose. Despite the longer treatment time, all patients tolerated SBRT treatment well. Typical treatment time ranged from 30 minutes to one hour. Patient comfort is a key factor. The patients were asked to continue their pain medications during SBRT as longer treatment time is expected. For some patients who presented with severe pain on a more urgent basis, conventional radiotherapy was started immediately for a few fractions to provide pain relief with SBRT, after which their pain was better controlled. Those patients were not included in this study. Full-course SBRT is preferred especially for patients with more radioresistant tumour, e.g. RCC, whereby higher fraction size may have positive impact on local tumour control.

### Treatment outcome

Thirteen of the 14 patients (93%) who received SBRT to extra-cranial metastases achieved significant pain relief. All the patients received narcotics analgesics prior to SBRT and they (except one) responded well to the treatment with significant pain relief and had declining need for narcotics analgesics. The only patient who did not have good pain relief had residual disease in the spine after surgery and radio-frequency ablation. His pain may have been due to a combination of residual disease and post-operative complications. The patients were observed to have achieved faster and more durable pain relief, sometimes even after one or two fractions of SBRT as compared to the conventional standard fractionation scheme. The patients who achieved significant pain relief also reported improvement in quality of life and a decrease in the use of pain medications. There was no significant (grade 2 or higher) treatment-related toxicity using RTOG/EORTC toxicity criteria observed in all patients.

**Figure 6:**
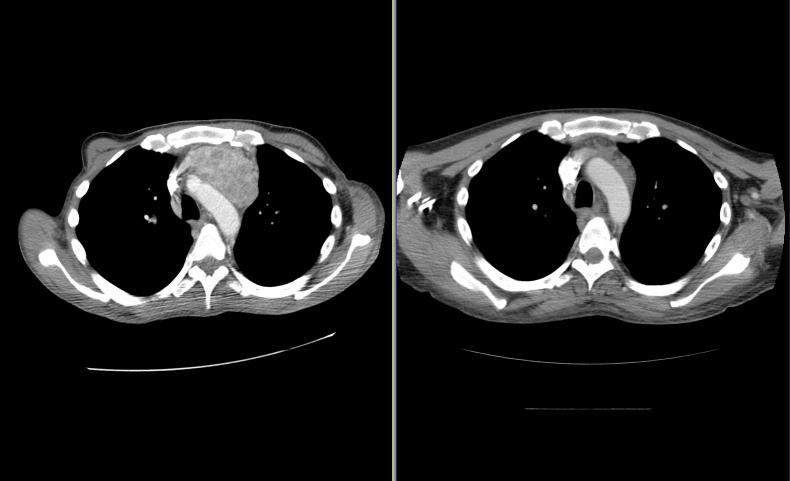
CT slice showing resolution of paratracheal/paraesophageal mass after image-guided SBRT. Left image is pre-treatment while right is post-treatment.

Follow-up imaging revealed excellent local control rates after SBRT. Figure 6 shows significant reduction of a solitary paratracheal/paraesophageal mass 6 weeks post SBRT in a patient with metastatic RCC. The patient’s symptoms of dysphagia and odynophagia also improved significantly. Local control is defined (RECIST criteria) as radiologically stable disease (SD), or partial response (PR) or complete response (CR). Two patients had local progression. There was no CR. The remaining patients had either PR or SD. The two local progressions occurred at one month and three months respectively post SBRT, yielding a local control rate of 87%. Target delineation was particularly difficult in one patient due to post-operative changes and “hardware placement” in the spine. Target volume was very large in another patient and the delivered dose was also limited by the adjacent critical structures of small bowels and spinal cord. In the two patients with primary RCC, tumour size remained unchanged but their pain improved and their renal function was unchanged post SBRT.

## DISCUSSION

RCC accounts for approximately 2% of all new cancer incidences worldwide. The incidence of RCC has been increasing steadily and may be due in part to better detection through increased use of CT or MRI imaging studies. Epidemiologic studies are still needed to identify the real reasons for this rise [8]. Nearly half the patients with RCC have metastatic disease on presentation or will have a recurrence [9]. Treatment of metastatic RCC is important. Recently, two multi-targeted tyrosine kinase inhibitors i.e. sunitinib and sorafenib, have been approved for treatment of metastatic RCC. Patients with metastatic RCC are living longer and thus local therapy such as radiotherapy has become more important especially in addressing symptomatic metastatic lesions.

RCC is traditionally thought to be a radio-resistant malignancy. It is believed that conventional radiotherapy does not have a role in the definitive management of RCC as there is no survival benefit of adding radiotherapy to the nephrectomy bed. Conventional radiotherapy has, however, been shown to be effective in palliating most sites of metastatic RCC including lung, bone and soft tissues in approximately 50% of patients [1]. On the other hand, SRS has been shown to provide a very high local control rate of up to 95% in various series [2-5]. This suggests that RCC may not be truly “radio-resistant” but more likely to be “radio-resistant” to lower fraction sizes. SBRT, as defined by the American Society of Therapeutic Radiology and Oncology and American College of Radiology practice guidelines as a “treatment method to deliver a high dose of radiation to the target, utilising either a single dose or a small number of fractions with a high degree of precision within the body” [10], is ideal to be utilised in patients with “radio-resistant” RCC. SBRT with the capability to deliver high dose per fraction, is made feasible by the recent refinement in precise IGRT and stereotaxis technology. In contrast to other local therapeutic modalities such as radio-frequency ablation, surgery and cryotherapy, SBRT offers the only non-invasive, highly efficient means of eradicating discreet tumour foci either at a primary or metastatic site. In addition, high ablative radiation dose has been shown to be effective in treating human RCC in animal models.[11]

This retrospective study further confirms the efficacy of SBRT in the treatment of metastatic RCC. Pain relief and local control were observed in 93% and 87% of patients, respectively. The results compare favorably to those using conventional radiotherapy [1] but are consistent with the more recent findings utilising SBRT for metastatic RCC. A local control rate of 90-98% was noted in a retrospective study involving 58 patients (50 patients with metastatic RCC and 8 patients with inoperable primary RCC) [12]. In another series of 48 patients with 60 RCC metastatic lesions involving various levels of spine, Gerszten and colleagues showed that pain was controlled in 89% of patients [13]. The two patients with medically inoperable primary RCC also did well with pain relief and stable disease on imaging. Beitler and colleagues also reported a series of nine patients with primary RCC treated with SBRT. There were four long-term survivors (minimum follow-up of 48 months) noted [14]. Similar results were seen in five patients with primary RCC treated with SBRT and had a follow-up of more than 4 years [12].

Essentially no significant treatment-related toxicity was noted in patients with metastatic RCC and primary RCC treated with SBRT in all reported series including the current report [12-14]. This is likely due to the precise delivery of high-dose radiation and the use of stereotactic and IGRT technology. Because of rapid fall-off in the isodose lines, only very limited normal tissues beyond tumour target received high-dose radiation. Also, as encouraging as very low toxicity in various organs, there was no deterioration of renal function including renal function tests and renal scans in patients whose primary RCC received SBRT. SBRT may offer a non-invasive nephron-sparing curative treatment modality for small RCC. SBRT may also play a role in patients with recurrent RCC in the remaining kidney, of which preservation of renal function is of utmost importance.

A few issues need to be mentioned and await further investigations. The response to SBRT was not seen clearly on conventional imaging such as CT images. Many patients showed complete pain relief and stable disease on CT in the follow-up. The same observation was also made by Wersall and colleagues [12]. Functional or molecular imaging with PET/CT with novel radio-pharmaceuticals may be more beneficial in assessing response to SBRT. The α/β of primary and metastatic RCC is not known. Ning and colleagues determined the α/β values for RCC cell lines A498 and Caki-1 to be 2.6 and 6.92, respectively[15] while DiBiase and colleagues used an α/β value of 10 for BED calculation. More work is required in order to determine the most appropriate α/β and BED values for RCC especially using the SBRT approach. Whether the use of SMART or SIB approach will have positive impact will also require further investigation.

The limitations of this study are the small number of patients and short follow up. Some patients have widely metastatic disease and the SBRT was used to treat the fastest growing lesion or the most symptomatic lesions. The short follow-up was also partly due to some of the patients having died from the systemic disease despite the local control of the SBRT-treated lesion. Despite all the patients having metastatic RCC, multiple sites were treated involving head and neck, chest, abdomen, bone and lungs, as well as the tumour masses of various sizes, contributing to a heterogenous group of patient population. Various fractionation schedules were used mainly because of the dose constraint placed on the surrounding normal structures. It is difficult to determine the best SBRT fractionation schedule because of the heterogeneity in the dose used. If hypoxia is one of the important factors determining “radioresistance” in RCC, longer fractionation schedules with moderate high dose fraction beyond 5-6 fractions may be better than 1-3 fractions with ultra-high dose fraction. Despite all the above-mentioned shortcomings, the initial experience has been encouraging. Prospective clinical trials are ongoing to address the best SBRT schedule for specified metastatic site and primary RCC in the affected kidney. Combining SBRT with novel targeted therapy such as tyrosine kinase inhibitors and mTOR inhibitors to maximize both local and systemic control is also planned.

## CONCLUSIONS

SBRT provides excellent local control and symptom palliation, without significant toxicity. SBRT may represent a novel non-invasive, nephron-sparing option for the treatment of primary RCC as well as extra-cranial metastatic RCC.

## ACKNOWLEDGEMENTS

This study is supported by a research grant from The Methodist Hospital Research Institute (TMHRI).
